# Role of C/EBP Homologous Protein (CHOP) and Nupr1 Interaction in Endoplasmic Reticulum Stress-Induced Apoptosis of Lens Epithelial Cells

**DOI:** 10.1007/s12033-024-01148-z

**Published:** 2024-05-21

**Authors:** Jinghua Li, Junyi Liu, Yongying Tang, Hong Zhang, Yuanping Zhang, Xu Zha, Xueying Zhao

**Affiliations:** Department of Ophthalmology, The Second Affiliated Hospital of Kunming Medical University, No.374 Yunnan-Burma Avenue, Wuhua District, Kunming, 650000 Yunnan China

**Keywords:** Cataract, Endoplasmic reticulum stress, C/EBP homologous protein, Nupr1, Lens epithelial cells

## Abstract

Our study mainly analyzed the mechanism of C/EBP homologous protein (CHOP) and its interacting protein Nupr1 on endoplasmic reticulum stress (ERS) induced lens epithelial cells (LEC) apoptosis. Cell proliferation was detected by CCK-8. Apoptosis was detected by flow cytometry and TUNEL. Nupr1 expression was detected by RT–qPCR. The expressions of CHOP, Nupr1, apoptosis-related protein, and ERS-related protein were detected by Western blot. DCFH-DA probe was used to detect cell ROS. The SOD, GSH-PX, and MDA contents were detected by the kit. Co-IP was used to detect the interaction between CHOP and Nupr1. The morphology of the lens was detected by HE staining. The result shows that Tunicamycin (TU) can induce endoplasmic reticulum stress and apoptosis in LEC in a concentration-dependent manner. TU induction leads to the occurrence of CHOP nuclear translocation. Overexpression of CHOP can further enhance the inhibitory effect of TU on LEC proliferation and the promotion of apoptosis, while knockdown of CHOP has the opposite effect. CHOP and Nupr1 are interacting proteins, and knockdown of Nupr1 or addition of Nupr1 inhibitor ZZW-115 can reverse the effects of TU and overexpression of CHOP, respectively. It has been observed in animal experiments that treatment with oe-CHOP can further aggravate the pathological lesions of the rat lens, while ZZW-115 can reverse the effect of oe-CHOP to a certain extent and improve the lesions of the rat lens. Overall, CHOP interacts with Nupr1 to regulate apoptosis caused by ERS and mediate cataract progression in rats, and this study provides a new potential therapeutic target for the treatment of cataract.

## Introduction

Cataract is an eye disease in which the lens becomes cloudy and less transparent due to a variety of reasons [1]. At present, the incidence and blindness rate of cataract are the most common eye diseases both at home and abroad, and they tend to occur in people of low socioeconomic status and developing countries [2, 3].Cataract not only affects the quality of life of patients, but also is an important factor leading to visual impairment. Data show that blindness caused by cataract accounts for 42% of all blind people [4]. Currently, the only effective treatment for cataracts is modern microsurgery, mainly through surgery to remove the affected lens and replace the intraocular lens [5]. However, surgery is expensive and there is a possibility of recurrence. In addition, there is no conventional drug treatment. Therefore, it is urgent to start from the molecular mechanism of cataract development to seek better cataract treatment.

Endoplasmic reticulum stress (ERS) refers to a pathophysiological process in which misfolded proteins accumulate excessively in the endoplasmic reticulum (ER) and that result in homeostasis imbalance and a series of cell dysfunction [6]. Normally, cellular immune damage is regulated by endoplasmic reticulum response to unfolded proteins (UPR), but at high levels of ERS, failure of the UPR pathway to eliminate misfolded proteins leads to apoptosis [7]. It has been reported that an important cause of cataract development is ERS-UPR induced apoptosis of lens epithelial cells (LEC) [8]. LEC is an important regulator of lens transparency and functional stability. Any injury of epithelial cells will lead to lens abnormalities, so the apoptosis of LEC becomes the cytological basis of cataract [9, 10]. Osnes-Ringen et al. found that epithelial cell apoptosis was associated with the formation of non-congenital cataracts [11]. Mulhern et al. found that in vitro cultivation of lens cells with high glucose could induce ERS and thus induce apoptosis of lens cells [12]. The role of excessive apoptosis of lens epithelium in cataract has been the focus of research in ophthalmology at home and abroad. To this end, we should keep up with the pace of scientific research.

C/EBP homologous protein (CHOP)/GADD153 as a specific endoplasmic reticulum transcription factor, CHOP/GADD153 be of great importance in ER stress-mediated apoptosis [13, 14]. Studies have shown that knockout of CHOP gene can lead to a decrease in the amount of apoptosis and the accumulation of misfolded or unfolded proteins [15, 16]. In addition, CHOP is closely related to apoptosis of lens epithelial cells. For example, knockdown of CHOP can effectively inhibit Krüppel-like factor 6 (KLF6)-induced LEC apoptosis [17]. During normal physiology, the expression of CHOP is generally at a low level, but when endoplasmic reticulum stress occurs, the expression level of CHOP increases due to the regulation of upstream transcription factors, thereby inducing apoptosis [18]. Currently, PERK/EIF-2a/ATF4 has been found to be a necessary upstream signaling pathway for CHOP-induced apoptosis, but its downstream mechanism after activation of CHOP by ERS is less studied. How CHOP interacts with downstream genes to mediate apoptosis in LEC is unclear.

Nupr1 is a stress-induced pleiotropic transcription factor that regulates physiological behaviors such as ferroptosis, autophagy, and apoptosis in a variety of diseases [19–21]. Other studies have shown that it also plays an important role in cellular stress and stress-related apoptosis [22]. Palam LR et al. found that PERK/eIF2-P/ATF4/CHOP pathway is involved in the transcriptional regulation of Nupr1 in pancreatic cancer cells [23]. It was reported that Nupr1 and CHOP can synergistically regulate the cell survival and apoptosis pathways of human chondrocytes, and the knockdown of Nupr1 has no effect on the expression of CHOP, but the knockdown of CHOP can mediate the expression of Nupr1 [24]. In many disease models, it was found that the expression of Nupr1 and CHOP increased simultaneously to promote the pathogenesis [25, 26]. Endoplasmic reticulum stress induced in melanoma cells also resulted in simultaneous increased expression of Nupr1 and CHOP [27]. These results suggest that Nupr1 and CHOP play an important role in the endoplasmic reticulum stress-induced apoptosis pathway, and CHOP has a potential upstream function of Nupr1 in this pathway.

Based on the association between CHOP and Nupr1 and ER stress, we hypothesized that Nupr1 is an important downstream interacting protein in ER stress activation of CHOP-induced apoptosis. Our study explored the role of CHOP and Nupr1 in endoplasmic reticulum stress-induced apoptosis of lens epithelial cells to determine their specific mechanisms in cataract pathogenesis.

## Materials and Methods

### Cell and Transfection

Human lens epithelial cells (HLECs) were obtained from Otwo Biotech (HTX2214, Shenzhen, China). The cells were cultured in an incubator at 37 ℃ and 5% CO_2_ with DMEM medium containing 10% fetal bovine serum and 1% streptomycin and penicillin. When the cells grew to about 70% ~ 80%, the cells were added with different concentrations of tunicamycin (0, 2.5, 5, 10 μg/ml) and incubated for 24 h. oe-CHOP, sh-CHOP, sh-Nupr1, and negative control (NC-oe, NC-sh) were transfected into HLECs using the Lipofectamine^TM^2000 transfection kit. In addition, the transfected oe-CHOP group was treated with Nupr1 inhibitor ZZW-115 (5 μM) for 6 h.

### CCK-8

Cell proliferation activity was detected with CCK-8 kit (CA1210, Solarbio, Beijing, China). HLECs (5 × 10^3^ cells/well) in logarithmic growth phase are seeded in 96-well plates. Cells were treated in different groups and cultured at 37 °C in a 5% CO_2_ incubator. According to the manufacturer’s instructions, 10μL CCK-8 solution was added at the corresponding time points and continued to be cultured in the incubator for 4 h. Finally, the absorbance was measured at 450 nm using an enzymoleter (ELX800, BioTeK, UK).

### TUNEL

TUNEL apoptosis assay kit (KGA7061, KeyGEN BioTECH, Nanjing, China) was used to detect HLECs apoptosis. In simple terms, HLECs are fixed with 4% paraformaldehyde for 30 min. After PBS rinsing, resuspend the cells with PBS containing 0.3% Triton X-100. After PBS rinsing, add 50 μL of TdT reaction solution to the sample and incubate at 37 °C for 60 min in the dark. Then, add 50 μL of Streptavidin-TRITC labeling solution dropwise to each sample and react at 37 °C in the dark for 30 min. Next, the nuclei were counterstained with DAPI staining solution and the reaction was carried out at room temperature for 15 min. Finally, the cells are observed under a fluorescence microscope.

### Apoptosis was Detected by Flow Cytometry

Apoptosis was detected using Annexin V-FITC apoptosis kit (C1062M, Beyotime, Shanghai, China). The treated HLECs of each group were collected, washed three times with PBS, and then the cells were re-suspended. At 25℃, add 5 μL Annexin V-FITC, gently mix, add 10 μL propyl iodide (PI) staining solution. Incubate at room temperature for 15 min away from light. Finally, flow cytometry (BD Biosciences, San Diego, CA, USA) was used to detect the percentage of apoptotic cells [28].

### RT-qPCR

TRIzol reagent (15596026, Invitrogen, USA) was used to extract total RNA. cDNA was synthesized using PrimeScript™ RT reagent Kit (RR037Q, Takara, Japan). Using cDNA as template, SYBR Green Master Mix (K0253, Life Technologies, USA) was used for qPCR. The reaction routine was as follows: pre-denaturation at 95℃ for 20 s; then, the amplification cycle was carried out at 95℃ for 1 s and 60℃ for 20 s, and there were 40 cycles in this stage. The internal reference gene was GAPDH, and the relative expression level was calculated by 2^−ΔΔCt^ method. Primers are shown in Table 1.

**Table 1:** Primer sequence

Gene	Forward sequence	Reverse sequence
Nupr1	5′-GACAGAGCTGGAGATGAGG-3′	5′-GCGGGAATAAGTCCTAGGG-3′
GAPDH	5′-TCAAGATCATCAGCAATGCC-3′	5′-CGATACCAAAGTTGTCATGGA-3′

### Western Blot

The tissue and cells were lysed by RIPA lysis solution (P0013B, Beyotime, Shanghai, China) to extract the protein. The protein concentration was detected by BCA reaction kit (P0012, Beyotime, Shanghai, China). Total proteins were isolated by SDS-PAGE gel electrophoresis, and the isolated proteins were transferred to polyvinylidene fluoride (PVDF) membranes (Millipore, USA). The membrane was sealed with 5% skim milk at room temperature for 1 h, and then incubated with primary antibody XBP-1 (1:1000, ab37152, Abcam, UK), ATF4 (1:1000, ab184909, Abcam, UK), GRP78 (1:1000, ab21685, Abcam, UK), Caspase-3 (1:5000, ab32351, Abcam, UK), PARP (1:1000, ab191217, Abcam, UK), Bax (1: 1000, ab32503, Abcam, UK), CHOP (1:2000, ab11419, Abcam, UK), Nupr1 (1:1000, ab161980, Abcam, UK) overnight at 4℃. The next day, secondary antibodies were added after washing the membrane and incubated for 1 h at room temperature in the dark. Colors were developed with an enhanced chemiluminescence (ECL) kit (Millipore, USA) and finally the bands were quantified using ImageJ software [29].

### Detection of MDA, GSH-PX, and SOD Content

Tissue or cell samples were collected, homogenized, or cleaved with PBS or cell lysate, and then supernatant was obtained by centrifugation. The content of corresponding indicators was detected according to the instructions of MDA kit (S0131S, Beyotime, Shanghai, China; BC0025, Solarbio, Beijing, China), SOD kit (A001-1, Nanjing Jiancheng Bioengineering Institute, Nanjing, China; BC5165, Solarbio, Beijing, China) kit, and GSH-Px kit (BC1175, Solarbio, Beijing, China; BC1195, Solarbio, Beijing, China).

### Detection of ROS Level

HLECs were seeded in 6-well plates. Add the diluted DCFH-DA (S0033S, Beyotime, Shanghai, China) and incubate in a 37 °C incubator for 20 min. After three washes of cells with serum-free cell culture, cell fluorescence was observed with fluorescence microscopy, and ROS levels of the cells were detected by flow cytometry.

### CHOP Nuclear Translocation was Observed by Immunofluorescence

HLECs cells were immobilized with 4% paraformaldehyde for 15 min, incubated in 0.5% Triton X-100 for 5 min, then block with 3% BSA for 30 min. Cells were incubated with CHOP antibodies (1:100, ab233045, Abcam, UK) for 1 h, and cells are rinsed and then incubated with FITC-conjugated secondary antibody for 1 h. Similarly, the cells were rinsed and the nuclei staining solution (DAPI) was added and stained at room temperature for about 5 min. The cells were observed by confocal microscopy (Leica-Microsystems, TCS SP8).

### Co-immunoprecipitation (Co-IP)

The LECs that overexpressed Flag-tagged Nupr1 and normal LECs were collected. Co-IP experiments were performed according to the instructions of the Pierce Classical Magnetic Bead IP/Co-IP Kit (88,804, Thermo Fisher Scientific, USA). Briefly, incubate the cell lysate with IP antibody (CHOP: MA1-250, Invitrogen, USA; anti-Flag: F1804, Sigma-Aldrich, USA) for 2 h at room temperature. Next, the antigen–antibody complex binds to Protein A/G magnetic beads for 1 h at room temperature. The beads are then washed twice with wash buffer and then once with purified water. Collect the beads to elute the antigen/antibody complex. Finally, Western blot was used to detect the expression of the experimental protein.

### Construction of Animal Models of Cataracts

SD rats are randomly divided into 4 groups, control group, Na_2_SeO_3_ group (cataract model), Na_2_SeO_3_ + oe-CHOP group, and Na_2_SeO_3_ + oe-CHOP + ZZW-115 group, 10 animals in each group. The control group was given subcutaneous injection of normal saline, and the model group was given sodium selenite (Na_2_SeO_3_) 20 μmol/kg subcutaneously on the nape of the neck, once every other day for three consecutive times. The Na_2_SeO_3_ + oe-CHOP group was given the same modeling method and oe-CHOP was injected through the tail vein. In the Na_2_SeO_3_ + oe-CHOP + ZZW-115 group, oe-CHOP and ZZW-115 (2.5 mg/kg) were injected once a day for 14 days. Six hours after the last dose, each group of rats was sacrificed with cervical dislocation, and the lens was peeled off from the eye and stored at -80 °C for backup.

### H&E Staining

After the paraffin sections are dewaxed, they are soaked in deionized water for 10 min, and then the sections are stained in hematoxylin staining solution for 10 min. After washing off the excess dyeing solution, the slices are placed in 1% hydrochloric acid alcohol solution, and when the slices turn light red, they are rinsed with running water to return to blue, and then the slices are stained in the eosin dye solution for about 90 s. The dehydrated sections were covered with neutral gum and observed under a microscope (Eclipse 80i, Nikon). Observe tissue morphology under a 20 × objective.

### Immunohistochemistry

Antigen retrieval is performed after paraffin section deparaffinization. It was then blocked with goat serum working solution at 37 °C for 10 min. Then add primary antibody CHOP (1:100, ab63392, Abcam, UK) and Nupr1 (1:200, ab234696, Abcam, UK) and incubate at 37 °C for 2 h. PBS rinsed with biotinylated secondary antibody and incubated at 37 °C for 30 min. After the DAB solution is developed, the sections are counterstained, dehydrated, transparent, and finally sealed. Finally, the positive cells were observed under microscope (Eclipse 80i, Nikon). Average optical density values were calculated using ImageJ-Pro Plus (Version 6.0, Media Cybernetics, USA) to determine the positive expression of CHOP and Nupr1.

### Statistical Analysis

Data analysis was performed using GraphPad Prism software (Version 8.0; La Jolla, USA). Data were expressed as mean ± standard deviation (SD). One-way analysis of variance was used for comparison between multiple groups, and student t test was used for comparison between two groups. P < 0.05 was considered statistically significant.

## Results

### Tunicamycin Induces Endoplasmic Reticulum Stress and Apoptosis in Lens Epithelial Cells

**Fig. 1:**
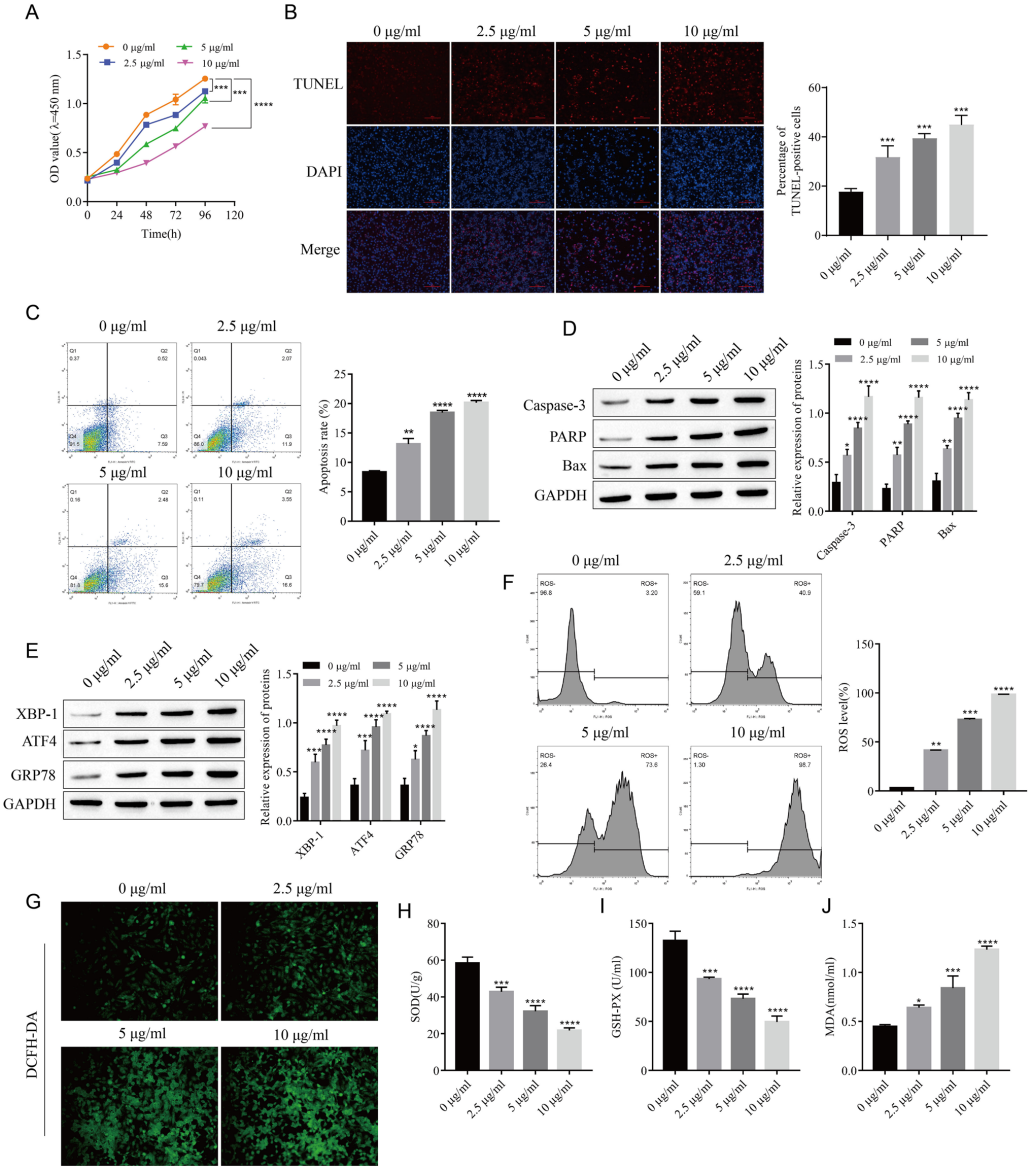
Tunicamycin induces endoplasmic reticulum stress and apoptosis in lens epithelial cells. **A** Cell proliferation was detected by CCK-8. **B** Apoptosis was detected by TUNEL. **C** Apoptosis was detected by flow cytometry. **D** The expressions of apoptosis-associated proteins Caspase-3, PARP, and Bax were detected by Western blot. **E** Western blot was used to detect the expressions of endoplasmic reticulum stress-related proteins XBP-1, ATF4, and GRP78. **F**–**G** Detection of ROS levels. **H**–**J** Analysis of SOD, GSH-PX, and MDA content. *P < 0.05, **P < 0.01, ***P < 0.001, ****P < 0.0001 vs. 0 μg/ml

Lens epithelial cells were treated with different concentration gradients of TU to observe the effects of TU on LEC apoptosis and ER stress. The proliferation of LECs was inversely correlated with TU concentration after CCK-8 detection (Fig. 1A). However, apoptosis was the opposite of proliferation (Fig. 1B, C). Western blot analysis of apoptosis-related proteins showed that the expressions of Caspase-3, PARP, and Bax were gradually upregulated with the increase of TU concentration (Fig. 1D). Western blot analysis of the expression of endoplasmic reticulum stress-related proteins also revealed that TU promoted the expression of XBP-1, ATF4, and GRP78 in a concentration-dependent manner (Fig. 1E). DCFH-DA detection of ROS levels, flow cytometry, and fluorescence microscopy results showed that ROS levels in LECs increased with increasing TU concentrations (Fig. 1F, G). After detection by the kit, SOD and GSH-PX levels decreased gradually, while MDA levels increased gradually (Fig. 1H–J). This indicated that TU could induce endoplasmic reticulum stress and apoptosis in LECs.

### TU-Induced Nuclear Translocation of CHOP Causes LEC Apoptosis

**Fig. 2:**
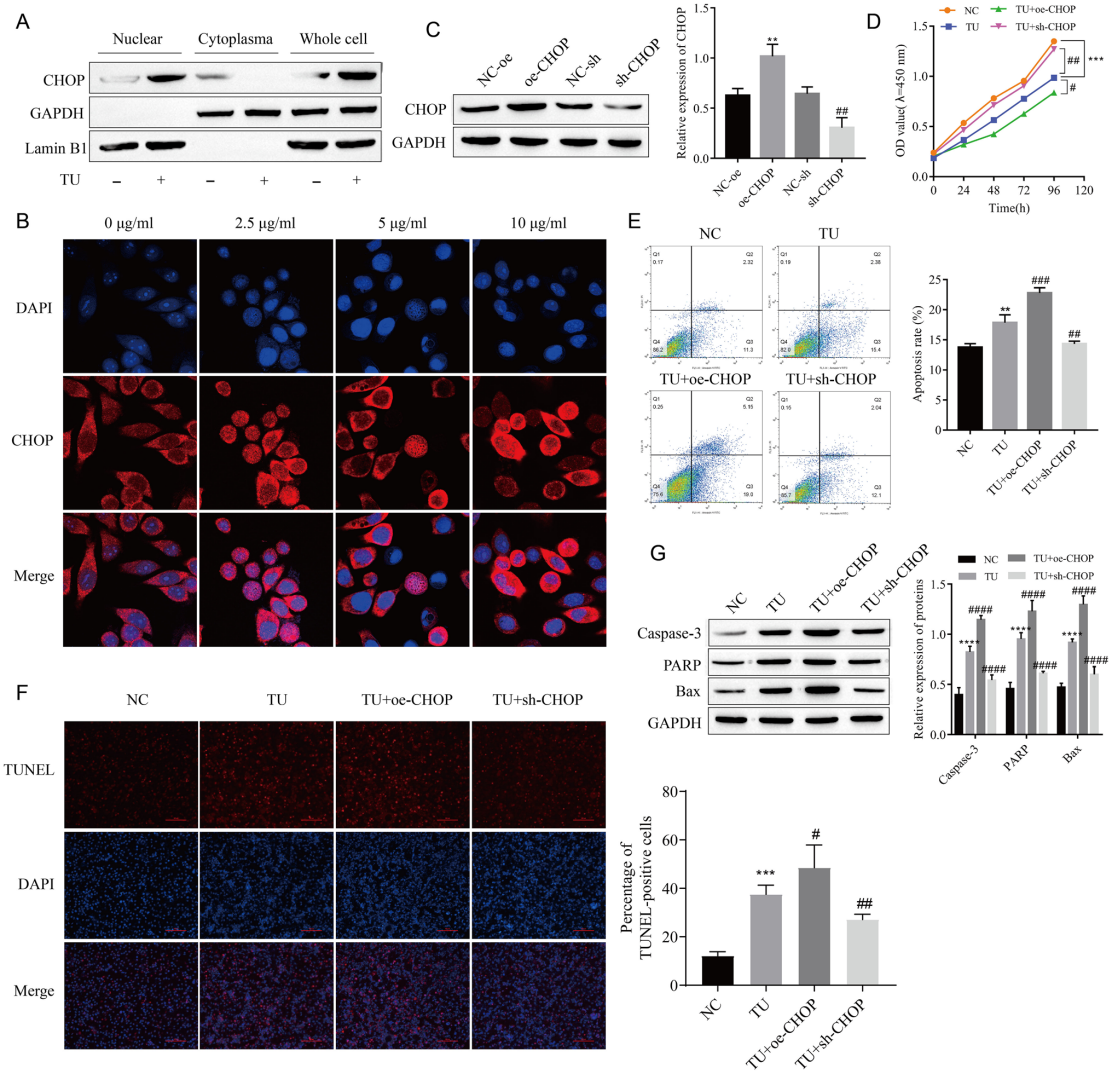
TU-induced nuclear translocation of CHOP causes LEC apoptosis. **A** Analysis of CHOP protein expression in nucleus and cytoplasm. **B** The nuclear translocation of CHOP was detected by confocal microscopy. **C** Western blot analysis of CHOP expression confirmed transfection efficiency. **D** Cell proliferation was detected by CCK-8. **E** Apoptosis was detected by flow cytometry. **F** Apoptosis was detected by TUNEL. **G** The expressions of apoptosis-related proteins Caspase-3, PARP, and Bax were detected by Western blot. **P < 0.01, ***P < 0.001, ****P < 0.0001 vs NC-oe or NC; ^#^*P* < 0.05, ^##^*P* < 0.01, ^###^*P* < 0.001, ^####^*P* < 0.0001 vs NC-sh or TU

After TU treatment of LECs, the expression of CHOP in the nucleus was observed. The results of Western blot showed that under normal LEC state, the expression of CHOP in the nucleus was very little, and the expression of CHOP in the nucleus increased significantly after TU induction (Fig. 2A). In addition, confocal microscopy was used to detect the nuclear translocation of CHOP, and the results showed that with the increase of TU concentration, the expression of CHOP in the nucleus gradually increased (Fig. 2B). oe-CHOP and sh-CHOP were transfected into LEC, and the transfection efficiency test showed that transfection was successful (Fig. 2C). The results of CCK-8 assay showed that the transfection of oe-CHOP could further promote the inhibition of TU on LEC proliferation, while the transfection of sh-CHOP had the opposite effect (Fig. 2D). Flow cytometry and TUNEL results showed that apoptosis was increased in the TU + oe-CHOP group and decreased in the TU + sh-CHOP group compared with the TU group (Fig. 2E, F). Western blot results also showed that overexpression of CHOP enhanced the promoting effect of TU on the expression of Caspase-3, PARP, and Bax proteins, while knockdown CHOP was the opposite result (Fig. 2G). This indicates that TU induces LEC apoptosis by promoting the nuclear expression of CHOP.

### TU-Induced Apoptosis is Dependent on Nupr1 Interacting with CHOP

**Fig. 3:**
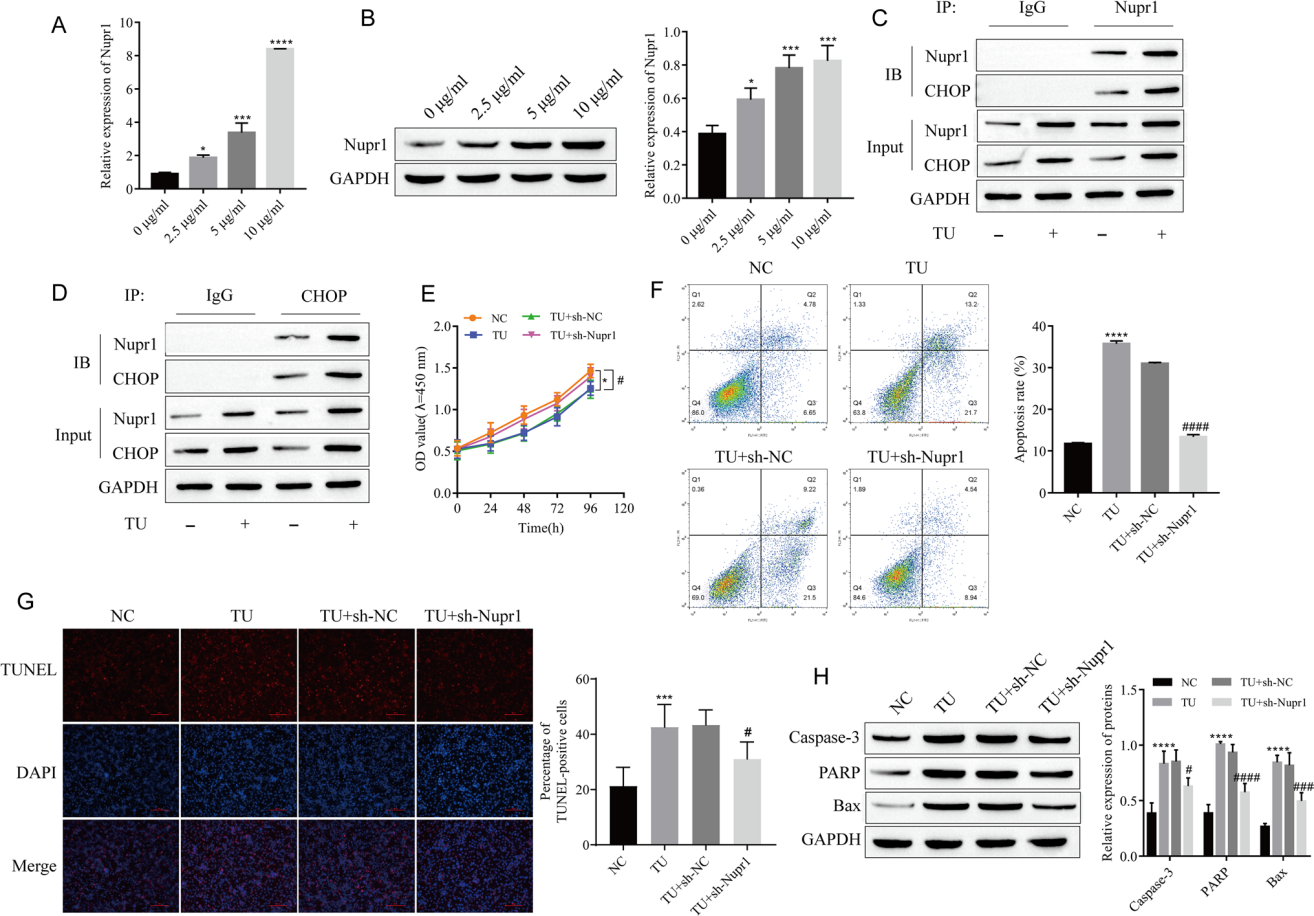
TU-induced apoptosis is dependent on Nupr1 interacting with CHOP. **A**, **B** Analysis of mRNA and protein expression of Nupr1. **C**, **D** The interaction between CHOP and Nupr1 was analyzed by Co-IP assay. **E** Cell proliferation was detected by CCK-8. **F**, **G** Apoptosis was detected by flow cytometry and TUNEL. **H** The expressions of apoptosis-associated proteins Caspase-3, PARP, and Bax were detected by Western blot. *P < 0.05, ***P < 0.001, ****P < 0.0001 vs 0 μg/ml or NC; ^#^*P* < 0.05, ^###^*P* < 0.001, ^####^*P* < 0.0001 vs TU-sh-NC

It was not difficult to find that the expression of Nupr1 gradually increased with the increase of TU concentration (Fig. 3A, B). Through co-immunoprecipitation experiments, we found that CHOP and Nupr1 are interacting proteins, and TU treatment can promote this effect (Fig. 3C, D). In addition, Nupr1 knockdown can effectively reverse the effect of TU on LEC proliferation (Fig. 3E). Flow cytometry and TUNEL detection of cell apoptosis showed that TU treatment increased cell apoptosis, and apoptosis was reversed after Nupr1 knockdown (Fig. 3F, G). At the same time, knockdown of Nupr1 could inhibit the promotion of Caspase-3, PARP, and Bax protein expression by TU (Fig. 3H). This indicates that TU-induced apoptosis is also dependent on Nupr1 interacting with CHOP.

### CHOP and Nupr1 Co-regulate Apoptosis Induced by ERS

**Fig. 4:**
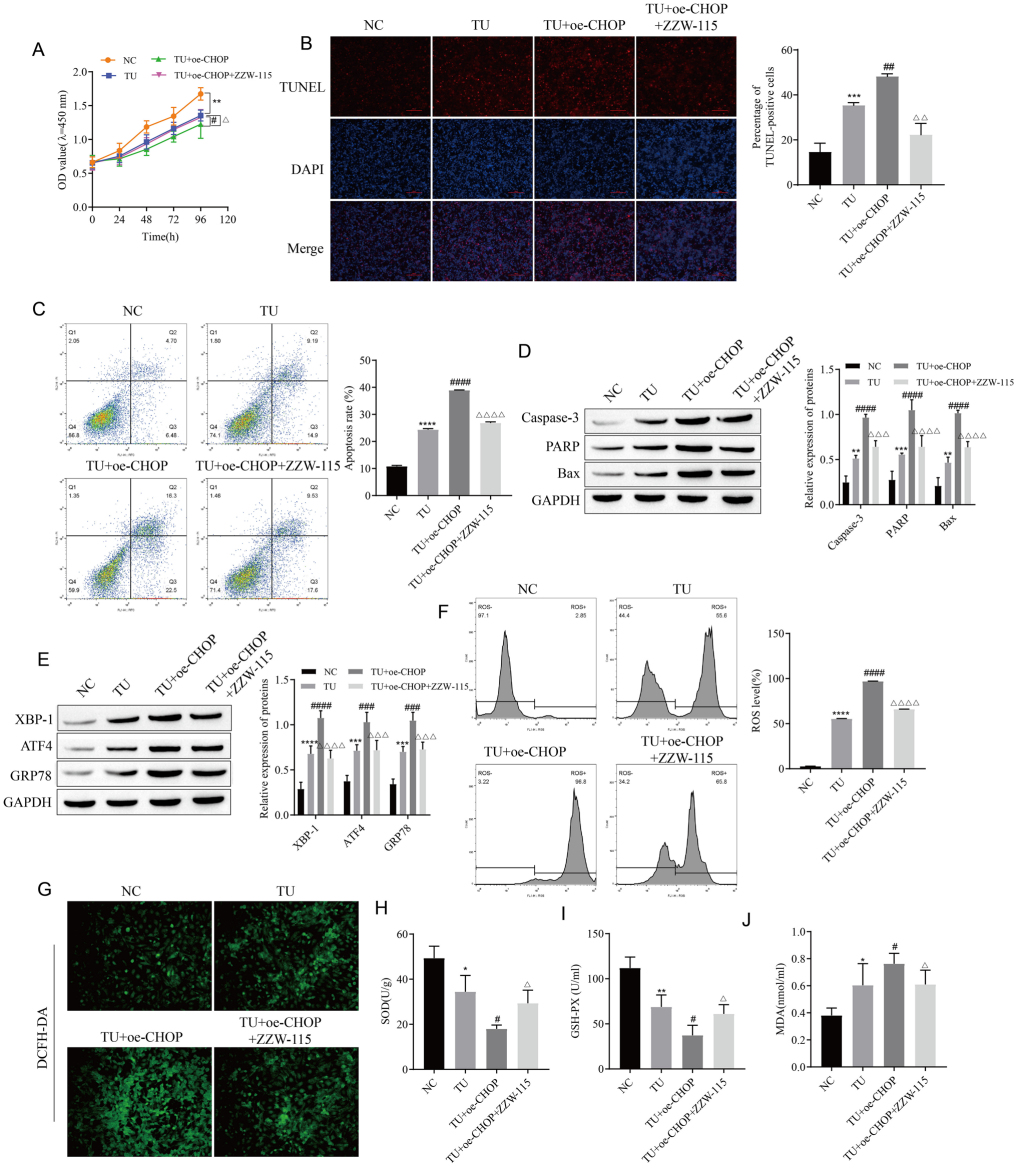
CHOP and Nupr1 co-regulate apoptosis induced by ERS. **A** Cell proliferation was detected by CCK-8. **B**, **C** Apoptosis was detected by TUNEL and flow cytometry. **D** The expressions of apoptosis-associated proteins Caspase-3, PARP, and Bax were detected by Western blot. **E** The expressions of endoplasmic reticulum stress-related proteins XBP-1, ATF4, and GRP78 were detected by Western blot. **F**–**G** Detection of ROS levels. **H**–**J** Analysis of SOD, GSH-PX, and MDA content. *P < 0.05, **P < 0.01, ***P < 0.001, ****P < 0.0001 vs NC; ^#^*P* < 0.05, ^##^*P* < 0.01, ^###^*P* < 0.001, ^####^*P* < 0.0001 vs TU; ^△^*P* < 0.05, ^△△^*P* < 0.01, ^△△△^*P* < 0.001, ^△△△△^*P* < 0.0001 vs TU + oe-CHOP

The oe-CHOP plasmid was transfected into LEC or the Nupr1 inhibitor ZZW-115 was added after transfection of oe-CHOP. The effects of TU on ERS and apoptosis were determined. CCK-8 results showed that overexpression of CHOP further promoted the inhibitory effect of TU on cell proliferation, but the addition of ZZW-115 could reverse this effect of CHOP (Fig. 4A). Flow cytometry and TUNEL detection of apoptosis revealed that the enhanced effect of overexpression of CHOP on TU-induced apoptosis was reversed by ZZW-115 (Fig. 4B, C). Similarly, the results of Western blot detection of apoptosis-related proteins showed that the enhanced effect of CHOP overexpression on TU-promoted Caspase-3, PARP, and Bax expression was reversed by treatment with Nupr1 inhibitor ZZW-115 (Fig. 4D). Compared with the TU + oe-CHOP group, the expressions of XBP-1, ATF4, and GRP78 were also down-regulated in the TU + oe-CHOP + ZZW-115 group (Fig. 4E). DCFH-DA detected ROS levels, showing that the enhanced effect of CHOP overexpression on TU-promoted ROS levels was reversed by ZZW-115 (Fig. 4F, G). In addition, ZZW-115 treatment increased SOD and GSH-PX levels after overexpression of CHOP, and decreased MDA levels (Fig. 4H–J). These results suggest that CHOP and Nupr1 co-regulate apoptosis induced by ERS.

### The Interaction Between CHOP and Nupr1 Affects the Progression of Cataract in Rats

**Fig. 5:**
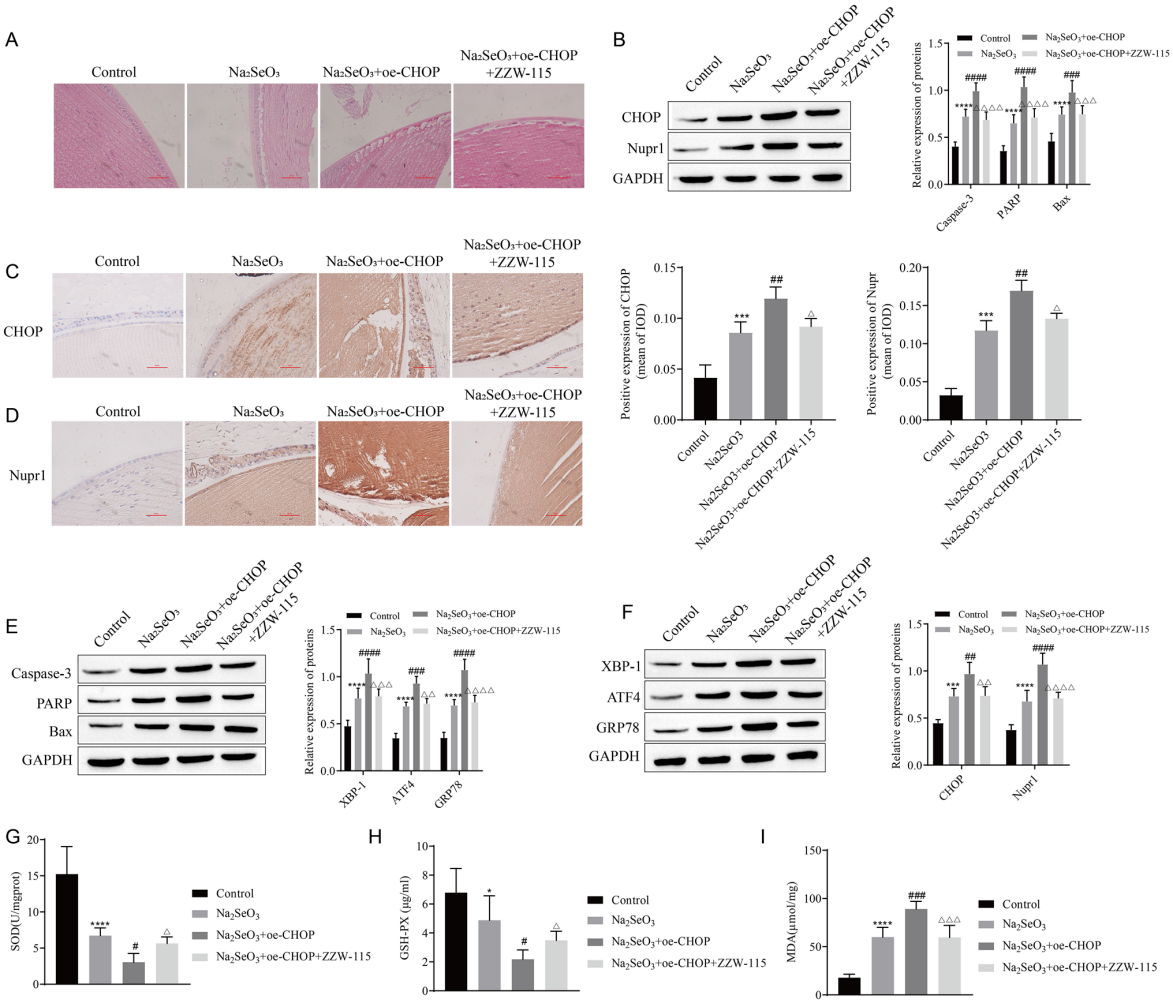
The interaction between CHOP and Nupr1 affects the progression of cataract in rats. **A** HE staining was used to detect the morphology of the lens. **B** The expressions of CHOP and Nupr1 were detected by Western blot. **C**, **D** Immunohistochemistry was used to detect the expression of CHOP and Nupr1. **E** The expressions of apoptosis-associated proteins Caspase-3, PARP, and Bax were detected by Western blot. **F** The expressions of endoplasmic reticulum stress-related proteins XBP-1, ATF4, and GRP78 were detected by Western blot. **G**–**I** The contents of SOD, GSH-PX, and MDA were detected by kit. *P < 0.05, ***P < 0.001, ****P < 0.0001 vs Control; ^#^*P* < 0.05, ^##^*P* < 0.01, ^###^*P* < 0.001, ^####^*P* < 0.0001 vs Na_2_SeO_3_; ^△^*P* < 0.05, ^△△^*P* < 0.01, ^△△△^*P* < 0.001, ^△△△△^*P* < 0.0001 vs Na_2_SeO_3_ + oe-CHOP

Na_2_SeO_3_ to build a cataract rat model. HE staining observed the lens of the rats. It can be seen that the lens epithelium of the control group is neatly arranged, and the fibrous lamellae are compact and regular, while the Na_2_SeO_3_-induced model group lens epithelium and fibrous lamellae are disorderly arranged, and vesicles and water fissures can be seen between the fibers, there are also a large number of vacuoles in the lens, and the treatment of oe-CHOP further promoted the pathological lesions of the rat lens, and the treatment of ZZW-115 could restore the pathological lesions aggravated by oe-CHOP to a certain extent (Fig. 5A). CHOP and Nupr1 expression in rat lens were detected. After being treated with Na_2_SeO_3_, the expressions of CHOP and Nupr1 were upregulated. CHOP and Nupr1 expressions were further upregulated after treatment of oe-CHOP on the basis of Na_2_SeO_3_ treatment, while the expressions of CHOP and Nupr1 were down-regulated in Na_2_SeO_3_ + oe-CHOP + ZZW-115 group compared with Na_2_SeO_3_ + oe-CHOP group (Fig. 5B–D). Detection of apoptosis and ERS-related proteins showed that the expression of Caspase-3, PARP, Bax, XBP-1, ATF4, and GRP78 were upregulated in the Na_2_SeO_3_ group, and the treatment of oe-CHOP further promoted the expression of these proteins, but ZZW-115 reversed the effect of oe-CHOP to some extent (Fig. 5E, F). Meanwhile, Na_2_SeO_3_ treatment decreased the expressions of SOD and GSH-PX in rat lens tissue, but increased the expression of MDA. Overexpression of CHOP promoted the effect of Na_2_SeO_3_, and the effect of oe-CHOP was reversed by ZZW-115 (Fig. 5G–I).

## Discussion

Severe cataracts can lead to blindness, reducing the burden of cataract remains a huge challenge [30]. Endoplasmic reticulum stress can induce the apoptosis of LEC [31], which in turn is closely related to the progression of cataract [32].Therefore, reducing the apoptosis of lens epithelial cells has a positive significance for preventing cataracts and delaying its progression. In this study, we investigated the role of CHOP, a key mediator of stress-induced apoptosis, in lens epithelial cell apoptosis, and found that CHOP can interact with Nupr1 to regulate endoplasmic reticulum stress-induced lens epithelial cells and mediate cataract progression in rats.

ERS has been shown to play a role in the progression of cataracts [33, 34]. When ERS occurs, UPR response is further activated, and PERK, IRE1, and ATF6, three important transmembrane proteins closely related to UPR, are activated after dissociation from GRP78/Bip and then play a role through signal transduction [35, 36]. The X-box binding protein 1 (XBP-1) translation product can promote the expression of UPR target molecules such as BiP/GRP78 containing endoplasmic reticulum stress response elements, so the upregulation of XBP-1 and GRP78 expression is often used as a marker of ERS [37, 38]. In addition, sustained high intensity of PERK activation can induce the overexpression of ATF4, thus activating CHOP to induce apoptosis [39]. In this study, the expression of endoplasmic reticulum stress-related proteins XBP-1, GRP78, and ATF4 was upregulated in lens epithelial cells treated with the ERS activator tunicamycin (TU), which is consistent with the previously reported results of TU-induced oxidative stress stimulation [40]. In addition, we found that TU treatment effectively inhibited the proliferation of LECs, promoted the occurrence of apoptosis of LECs, and upregulated the expression of apoptosis-related proteins Caspase-3, PARP, and Bax. Bax (Bcl-2-associated X protein) is one of the members of the Bcl-2 protein family, which can promote apoptosis in the form of dimers with proteins of the same family, and Caspase-3 and PARP have also been used as indicators for apoptosis detection [41]. In this study, we found that the induction of apoptosis by TU was consistent with that of Abhari BA et al. [42]. It was further determined that TU induced the occurrence of apoptosis of LECs through endoplasmic reticulum stress.

CHOP is an important regulator of ERS-mediated apoptosis [43]. McCullough et al. showed that the increase in CHOP expression led to the down-regulation of the expression of the anti-apoptotic protein Bcl-2 gene, which quickly triggered apoptosis after disrupting redox homeostasis [44]. CHOP also directly dephosphorylates eIF2α by activating the DNA-damaging protein GADD34, thereby increasing ERS and apoptosis [45, 46]. In addition, studies have shown that CHOP translocation to the nucleus is closely related to its mediated apoptotic function [47, 48]. Our study found that TU can promote the expression of CHOP in the nucleus. Overexpression of CHOP enhanced the promotion of TU on the expression of pro-apoptotic proteins in LECs, while knockdown of CHOP was the opposite. This is consistent with previously reported studies on CHOP inducing ER stress-mediated apoptosis in a Bax pathway-dependent manner [49].

Nupr1 is a key player in cellular stress. Nupr1-mediated ERS can affect the apoptotic process of vascular endothelial cells [50]. It has also been reported that Nupr1 is involved in the autophagy process of intestinal epithelial cells through ERS [51]. In addition, Nupr1 has been found to play an important role in the expression of TRB3 under CHOP-induced RES in human tumor cells [52]. Our study found that CHOP and Nupr1 are interacting proteins, and the addition of Nupr1 inhibitor ZZW-115 can reverse the promoting effect of CHOP on the apoptosis of LECs, and also to some extent improve the aggravation of pathological changes in cataract rats induced by overexpression of CHOP.

## Conclusion

**Fig. 6:**
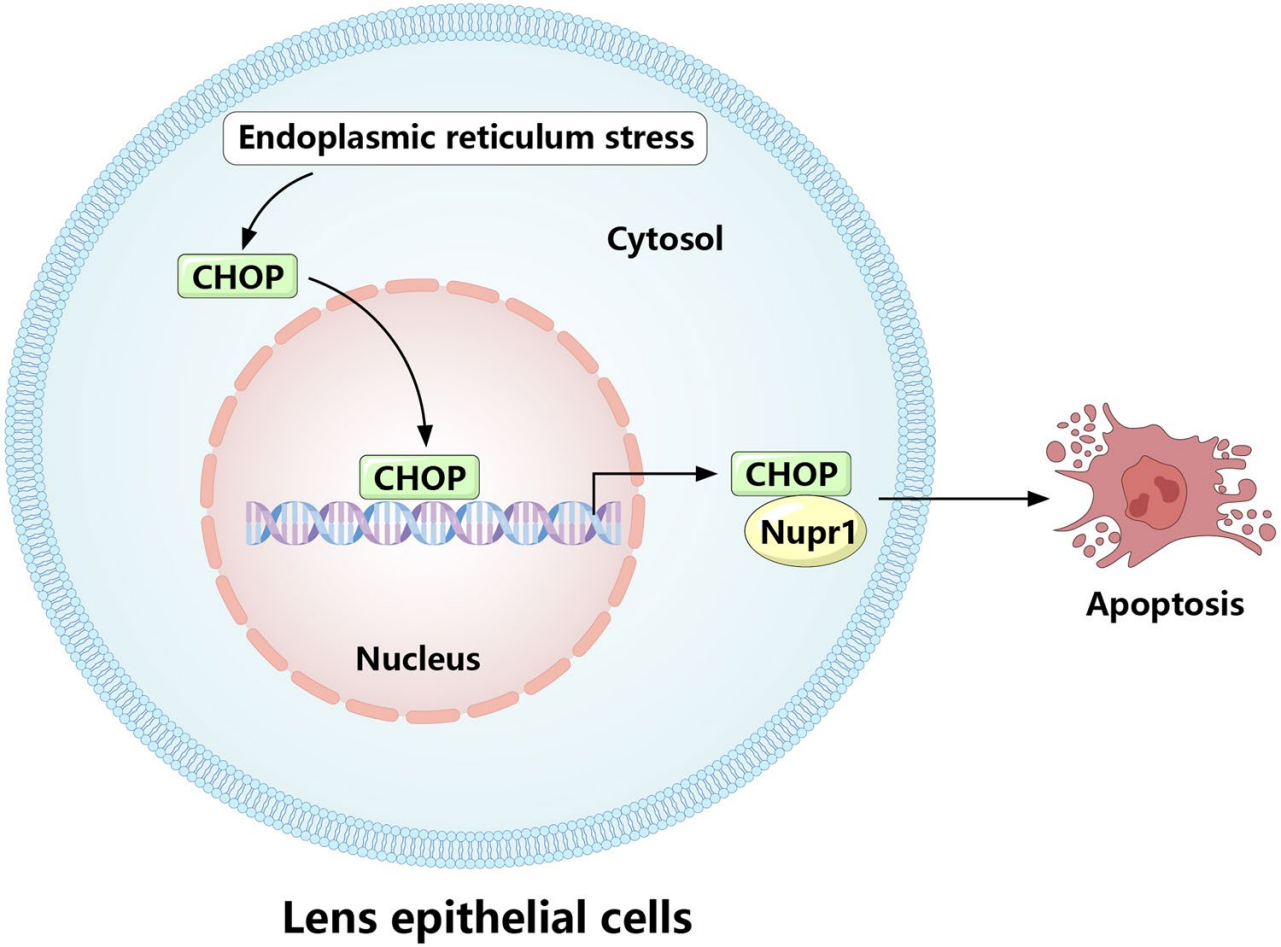
Diagram of the mechanism of CHOP and Nupr1 interaction in endoplasmic reticulum stress-induced apoptosis of lens epithelial cells

Overall, our study found that the expression of CHOP and Nupr1 was upregulated in the state of endoplasmic reticulum stress. CHOP and Nupr1 can co-regulate the apoptosis of LECs caused by endoplasmic reticulum stress, thereby affecting the progression of cataract in rats (Fig. 6). This provides an important potential target for the study of new treatments for cataracts.

## Data Availability

The datasets used and/or analyzed during the current study are available from the corresponding author upon reasonable request.
